# Saturation Effect of Body Mass Index on Bone Mineral Density in Adolescents of Different Ages: A Population-Based Study

**DOI:** 10.3389/fendo.2022.922903

**Published:** 2022-07-05

**Authors:** Yujuan Ouyang, Yingping Quan, Chengyi Guo, Songlin Xie, Changxiong Liu, Xiongjie Huang, Xinfeng Huang, Yanming Chen, Xiangjun Xiao, Nengqian Ma, Ruijie Xie

**Affiliations:** ^1^Nuclear Industry Health School, The Affiliated Nanhua Hospital, Hengyang Medical School, University of South China, Hengyang, China; ^2^Department of Gastrointestinal Surgery, The Affiliated Nanhua Hospital, Hengyang Medical School, University of South China, Hengyang, China; ^3^Department of Hand & Microsurgery, The Affiliated Nanhua Hospital, Hengyang Medical School, University of South China, Hengyang, China; ^4^Department of Hepatobiliary Surgery, The Affiliated Nanhua Hospital, Hengyang Medical School, University of South China, Hengyang, China

**Keywords:** bone mineral density, osteoporosis, NHANES, obese, body mass index, adolescent

## Abstract

**Background:**

Adolescence is a critical period for bone development, and peak bone mass may be reached in late adolescence. Boosting bone accumulation at this time can help preserve adult bone health and avoid osteoporosis later in life. Body mass index (BMI) has been found to have a favorable impact on bone mineral density (BMD) in previous research. However, excessive obesity is harmful to health and may lead to various systemic diseases. Therefore, finding an appropriate BMI to maintain a balance between obesity and BMD is critical for adolescents.

**Methods:**

The datasets from the National Health and Nutrition Examination Survey (NHANES) 2011–2020 were used in a cross-sectional investigation. Multivariate linear regression models were used to examine the linear connection between BMI and BMD. Fitted smoothing curves and threshold effect analysis were used to describe the nonlinear relationship. Subgroup analyses were then conducted based on gender and age.

**Results:**

This population-based study included a total of 6,143 adolescents aged 8–19 years. In a multivariate linear regression analysis, a good association between BMI and total BMD was shown [0.014 (0.013, 0.014)]. This positive association was maintained in all subgroup analyses grouped by sex and age. Furthermore, the association between BMI and BMD was nonlinear with a saturation point present, as evidenced by smoothed curve fitting. According to the threshold effect study, with an age group of two years, adolescents of different ages had different BMI saturation values with respect to BMD.

**Conclusions:**

Our study showed a significant positive and saturated association between BMI and BMD in adolescents aged 8–19 years. Maintaining BMI at saturation values may reduce other adverse effects while achieving optimal BMD.

## Background

Osteoporosis is a long-term disorder marked by reduced bone mineral density (BMD) that affects a huge number of people (1). Adolescence is a critical period for bone development, and peak bone mass (PBM) may be reached in late adolescence (2, 3). There is evidence that increasing PBM by 5% throughout childhood and adolescence reduces the risk of osteoporotic fractures by 40%, whereas increasing PBM by 10% reduces the risk by half (4, 5). As a result, boosting bone accumulation at this time can help preserve adult bone health and avoid osteoporosis later in life (6, 7). In addition to metabolic disorders such as lipids (8, 9), serum calcium (10), and non-alcoholic fatty liver disease (11), obesity has been shown to have an impact on adolescent BMD (12). Meanwhile, scientists are working to discover novel ways to prevent and treat osteoporosis.

Obesity is a major health issue that affects individuals all over the globe (13). The prevalence of overweight and obesity among children and adolescents aged 5–19 years rose sharply from 4% in 1975 to more than 18% in 2016 (14). Previous research has shown that body mass index (BMI) and BMD have a significant positive relationship (15, 16). However, excessive obesity not only has very serious consequences for various organs and systems (17, 18) but may also increase the risk of fractures in children (19). We hypothesized that BMI had a saturation point, and that keeping BMI at this level would provide the greatest balance between obesity and BMD. Therefore, finding an appropriate BMI to maintain a balance between obesity and BMD is critical for adolescents. As a result, we examined the relationship between BMI and BMD in adolescents in this study, utilizing a large sample of people aged 8–19 from the National Health and Nutrition Examination Survey (NHANES).

## Methods

### Study Population

**Figure 1:**
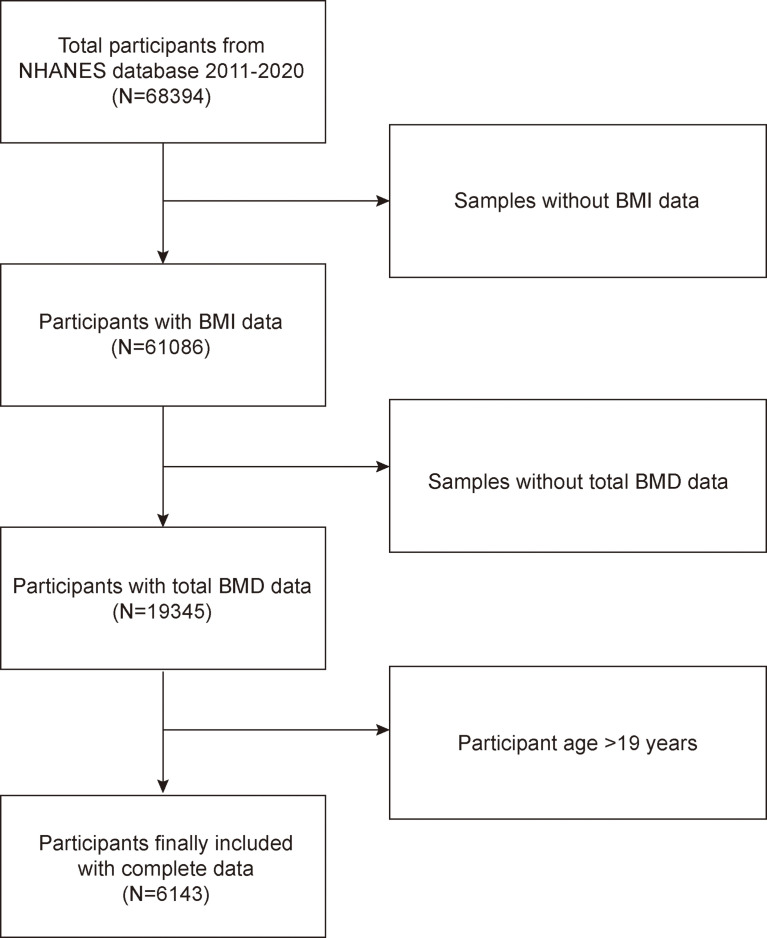
Flow chart of participants selection. NHANES, National Health and Nutrition Examination Survey; BMD, bone mineral density; Body Mass Index, BMI.

The National Health and Nutrition Examination Survey is a representative survey of the US national population that uses a complicated, multistage, and probabilistic sampling methodology to provide a wealth of information about the general US population’s nutrition and health (20). The 2011–2020 continuous cycle of the US NHANES dataset was used for this investigation. In this round, there were 68,394 participants. After eliminating patients who lacked information on laboratory and demographic characteristics, a total of 6,143 subjects were included in the analysis. **Figure 1** illustrates the sample selection flow chart.

### Study Variables

The dependent variable in this study is BMI, with total BMD as the intended independent variable. Weight divided by height squared is how BMI is computed according to the international guidelines. To ensure that the data are trustworthy, outliers will be subjected to appropriate scrutiny. The age, weight, height, and gender of the individual are used to verify the accuracy of the data. After verification, inaccurate data were removed. A dual-energy X-ray absorptiometry scan was used to calculate the total BMD. Covariates included age, gender, race, standing height, education level, family income-to-poverty ratio, activities status, diabetes status, alanine transaminase (ALT), weight, alkaline phosphatase (ALP), waist circumference, aspartate aminotransferase (AST), total calcium, total cholesterol, direct high-density lipoprotein (HDL) cholesterol, low-density lipoprotein (LDL) cholesterol, triglyceride, phosphorus, blood urea nitrogen, and serum glucose. For more detailed information on BMI, total BMD, and confounders, visit http://www.cdc.gov/nchs/nhanes/.

### Statistical Analysis

The statistical study was carried out using the statistical computing and graphics software R (version 4.1.3), Origin (version 2021b), and EmpowerStats (version 2.0). Baseline tables for the study population were statistically described by BMI subgroup, and continuous variables are described using means ± standard deviation and weighted linear regression models. The beta values and 95% confidence intervals (CI) were calculated using multivariate linear regression analysis between the BMI and BMD. The multivariate test was built using three models: Model 1: no variables adjusted; Model 2: gender, age, and race adjusted; Model 3: adjusted for all covariates except for height and weight, which had a large effect on exposure factors. By adjusting the variables, smoothed curve fits were done simultaneously. A threshold effects analysis model was used to examine the relationship and saturation value between BMI and BMD. Finally, the same statistical study methods described above were conducted for the gender and BMI subgroups. It was determined that P < 0.05 was statistically significant. We used a weighting approach to reduce the significant volatility of our dataset.

## Results

### Baseline Characteristics

**Figure 2:**
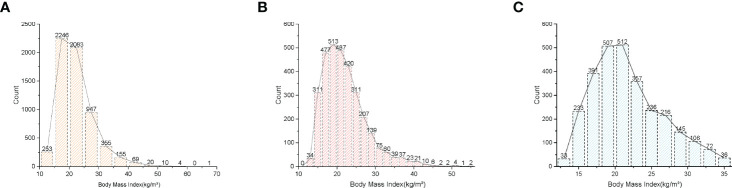
Distribution histogram of BMI. **(A)** Among all participants. **(B)** Among all males. **(C)** Among all females. Body Mass Index, BMI.

A total of 6,143 adolescents were included in this study based on the inclusion and exclusion criteria, and the average age of the participants was 13.10 ± 3.46 years. Among these participants, 51.77% were boys, 48.23% were girls, 27.53% were non-Hispanic white, 23.98% were non-Hispanic black, 20.56% were Mexican-American, and 27.93% were other races. The mean (SD) concentrations of BMI and total BMD were 22.28 (5.99) kg/m^2^ and 0.95 (0.16) g/cm^2^, respectively. **Table 1** lists the clinical features of the study participants, and column stratified grouping was based on BMI dividing all participants equally into four groups by number. **Figure 2** shows the frequency distribution of BMI for total participants and for participants of different genders. In comparison to the bottom quartile, those in the top quartile with higher BMI were more likely to be females and older, with a higher proportion of non-Hispanic blacks and Mexican-Americans, with higher prevalence of diabetes, and with higher levels of weight, standing height, waist circumference, AST, ALT, Total cholesterol, LDL cholesterol, serum glucose, total BMD, triglyceride, and total BMD but with lower levels of ratio of family income to poverty, ALP, total calcium, direct HDL cholesterol, phosphorus, and blood urea nitrogen (P < 0.05).

**Table 1:** Characteristics of the participants.

Outcome	BMI (kg/m^2^) Quartiles				*P*-value
	Q1, 18.1< (N = 1,524)	Q2, 18.1-21.0 (N = 1,532)	Q3, 21.1-23.4 (N = 1,549)	Q4, >23.4 (N = 1,538)	
Age (years)	10.406 ± 2.425	13.053 ± 3.233	14.206 ± 3.286	14.699 ± 3.128	<0.001
Gender (%)					<0.001
Male	55.381	48.825	53.777	49.090	
Female	44.619	51.175	46.223	50.910	
Race (%)					<0.001
Non-Hispanic White	31.234	27.350	28.018	23.537	
Non-Hispanic Black	23.031	24.086	21.821	26.983	
Mexican-American	16.929	18.277	21.562	25.423	
Other race	28.806	30.287	28.599	24.057	
Weight (kg)	33.496 ± 7.889	47.850 ± 9.186	59.129 ± 10.108	81.402 ± 19.604	<0.001
Standing height (cm)	142.756 ± 13.682	155.671 ± 14.374	160.363 ± 12.984	162.745 ± 11.633	<0.001
Waist circumference(cm)	60.464 ± 4.989	70.018 ± 4.380	78.462 ± 5.098	96.614 ± 13.295	
Ratio of family income to poverty	2.270 ± 1.664	2.147 ± 1.577	2.110 ± 1.577	1.838 ± 1.394	<0.001
*Moderate activities (%)*					0.133
Yes	52.742	51.949	50.442	50.485	
No	47.258	48.051	49.558	49.515	
*Diabetes status (%)*					<0.001
Yes	0.000	0.196	0.646	0.845	
No	100.000	99.804	99.364	99.155	
ALT (U/L)	16.027 ± 5.400	16.173 ± 7.555	18.196 ± 9.688	23.701 ± 17.523	<0.001
AST (U/L)	24.545 ± 6.062	23.028 ± 6.887	23.608 ± 9.358	24.823 ± 15.572	0.002
ALP (U/L)	201.910 ± 105.025	150.348 ± 106.815	118.515 ± 82.951	109.776 ± 69.877	<0.001
Total calcium (mmol/L)	2.415 ± 0.074	2.407 ± 0.073	2.400 ± 0.073	2.386 ± 0.079	<0.001
Total cholesterol (mmol/L)	4.075 ± 0.733	3.990 ± 0.672	4.043 ± 0.743	4.164 ± 0.804	<0.001
Direct HDL cholesterol (mmol/L)	1.548 ± 0.337	1.450 ± 0.308	1.353 ± 0.300	1.207 ± 0.270	<0.001
LDL cholesterol (mmol/L)	2.059 ± 0.575	2.121 ± 0.619	2.282 ± 0.689	2.422 ± 0.723	<0.001
Triglyceride(mmol/L)	0.677 ± 0.366	0.716 ± 0.389	0.806 ± 0.517	1.088 ± 0.702	<0.001
phosphorus(mmol/L)	1.536 ± 0.214	1.445 ± 0.223	1.383 ± 0.206	1.356 ± 0.203	<0.001
Blood urea nitrogen(mmol/L)	3.857 ± 1.289	3.883 ± 1.204	4.014 ± 1.232	3.826 ± 1.129	0.003
Serum glucose(mmol/L)	4.913 ± 0.537	4.891 ± 0.770	4.853 ± 0.586	5.016 ± 0.746	<0.001
Body Mass Index(kg/m²)	16.199 ± 1.210	19.558 ± 0.849	22.825 ± 1.135	30.467 ± 5.324	<0.001
Lumbar bone mineral density(g/cm^2^)	0.708 ± 0.116	0.855 ± 0.166	0.922 ± 0.190	0.968 ± 0.184	<0.001
Total bone mineral density(g/cm^2^)	0.968 ± 0.184	0.932 ± 0.130	1.000 ± 0.143	1.042 ± 0.140	<0.001

Mean ± SD for continuous variables: P-value was calculated by weighted linear regression model. % for categorical variables: P-value was calculated by weighted chi-square test.

### Association Between BMI and Total BMD

**Table 2** shows the results of the multivariate regression analysis. In the unadjusted model [0.014 (0.013, 0.014)], BMI was highly associated with total BMD. In addition, this relationship remained significant after adjusting corresponding variables in Model 2 [0.006 (0.005, 0.006)] and Model 3 [0.005 (0.004, 0.005)]. In the unadjusted model, the beta value was 0.014, meaning that, for every unit increase in BMI, the total BMD increased by 0.014 g/cm^2^.

**Table 2:** Association between BMI (kg/m²) and total bone mineral density (g/cm^2^).

	Model 1β (95*%* CI) *P-*value	Model 2β (95*%* CI) *P-*value	Model 3β (95*%* CI) *P-*value
Body mass index(kg/m²)	0.014 (0.013, 0.014) <0.001	0.006 (0.005, 0.006) <0.001	0.005 (0.004, 0.005) <0.001
Subgroup analysis stratified by gender			
Males	0.015 (0.014, 0.015) <0.001	0.005 (0.005, 0.006) <0.001	0.005 (0.003, 0.006) <0.001
Females	0.013 (0.012, 0.014) <0.001	0.006 (0.005, 0.006) <0.001	0.004 (0.003, 0.006) <0.001
Subgroup analysis stratified by age			
8–9 years (n = 1,223)	0.007 (0.006, 0.008) <0.001	0.007 (0.006, 0.008) <0.001	0.007 (0.006, 0.008) <0.001
10–11 years (n = 1,180)	0.007 (0.006, 0.008) <0.001	0.007 (0.006, 0.008) <0.001	0.007 (0.006, 0.008) <0.001
12–13 years (n = 944)	0.007 (0.006, 0.008) <0.001	0.006 (0.005, 0.007) <0.001	0.006 (0.004, 0.008) <0.001
14–15 years (n = 959)	0.007 (0.006, 0.008) <0.001	0.007 (0.006, 0.008) <0.001	0.006 (0.004, 0.007) <0.001
16–17 years (n = 961)	0.004 (0.003, 0.005) <0.001	0.004 (0.004, 0.005) <0.001	0.004 (0.003, 0.005) <0.001
18–19 years (n = 876)	0.004 (0.002, 0.005) <0.001	0.003 (0.002, 0.004) <0.001	0.004 (0.002, 0.006) <0.001
Subgroup analysis stratified by BMI			
Q1, 18.1<	0.040 (0.036, 0.044) <0.001	0.022 (0.019, 0.025) <0.001	0.020 (0.016, 0.024) <0.001
Q2, 18.1-21.0	0.032 (0.024, 0.039) <0.001	0.014 (0.010, 0.019) <0.001	0.014 (0.009, 0.018) <0.001
Q3, 21.1–23.4	0.014 (0.008, 0.020) <0.001	0.007 (0.004, 0.011) <0.001	0.012 (0.004, 0.020) 0.002
Q4, >23.4	0.005 (0.004, 0.006) <0.001	0.001 (0.000, 0.002) <0.001	0.001 (0.001, 0.002) 0.042
P for trend	<0.001	<0.001	<0.001

Model 1: No covariates were adjusted. Model 2: Age, gender, and race were adjusted. Model 3: Age, gender, race, education level, ratio of family income to poverty, activities status, diabetes status, ALT, ALP, AST, total calcium, total cholesterol, direct HDL cholesterol, LDL cholesterol, triglyceride, phosphorus, blood urea nitrogen, and serum glucose were adjusted. *In the subgroup analysis stratified by gender or race, the model is not adjusted for the stratification variable itself.

In all subgroups, BMI showed a significant positive association with total BMD. In the subgroup analysis stratified by sex, the effect values were closer for boys and girls, 0.015 and 0. 013, respectively. Whereas in the subgroup analysis stratified by age, the effect values for adolescents aged 8–15 years were significantly larger than those for adolescents aged 16–19 years, implying that, for each unit increase in BMI for adolescents aged 8–15 years, BMD increased by 0.07 g/cm^2^ and, for each unit increase in BMI for adolescents aged 16–19 years, BMD increased by only 0.04 g/cm^2^. In addition, the results of the BMI quartile subgroup analysis showed that there was a dose-response relationship between BMI and total BMD.

### Non-Linearity and Saturation Effect Analysis Between BMI and Total BMD

**Figure 3:**
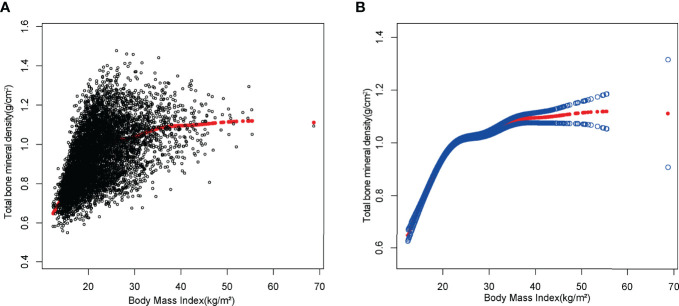
The association between BMI and total bone mineral density. **(A)** Each black point represents a sample. **(B)** The solid red line represents the smooth curve fit between variables. Blue bands represent the 95% confidence interval from the fit.

**Figure 4:**
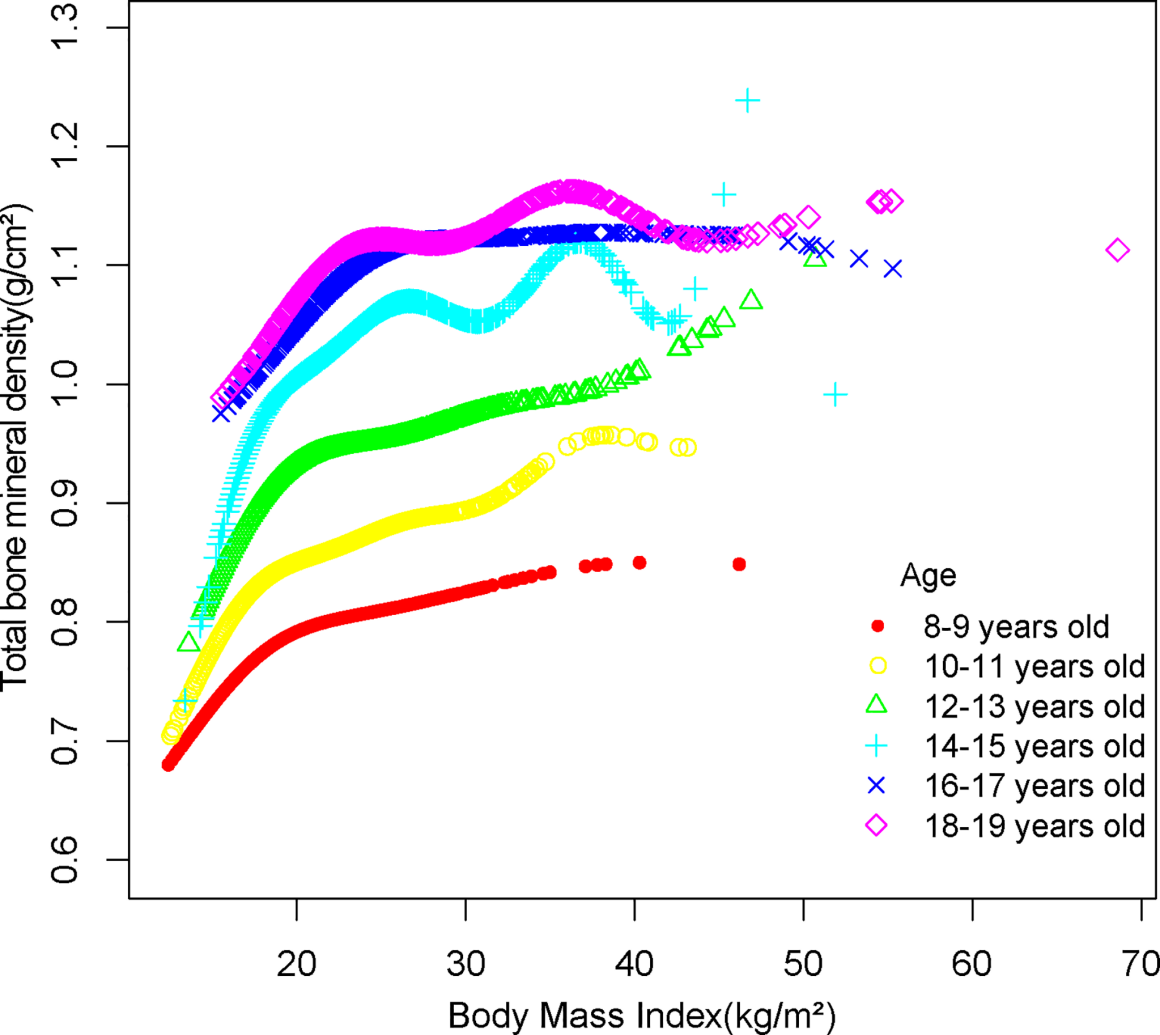
The association between BMI and total bone mineral density stratified by age.

Smooth curve fittings were performed to characterize the non-linear relationship and saturation effect between BMI and total BMD (**Figures 3**, **4**). We discovered that the saturation effect value between BMI and total BMD was 21.5 kg/m^2^ in total participants (**Table 3**). When the BMI was under 21.5 kg/m^2^, the effect value was 0.036. However, when BMI exceeded 21.5 kg/m^2^, the effect value became 0.005. All participants were divided into six groups according to an age group of 2 years and the saturation values of BMI for total BMD of adolescents at different ages were determined using smoothed fitted curves and saturation effects analysis (**Table 3**).

**Table 3:** Saturation effect analysis of BMI (kg/m^2^) on total BMD (g/cm^2^).

Total bone mineral density	Model: saturation effect analysis
BMI turning point (K),kg/m^2^	21.5
<K, effect 1	0.036 (0.034, 0.037) <0.001
>K, effect 2	0.005 (0.004, 0.006) <0.001
Subgroup analysis stratified by age	
BMI turning point for 8–9 years old(K),kg/m^2^	16.9
<K, effect 1	0.023 (0.019, 0.027) <0.001
>K, effect 2	0.004 (0.003, 0.005) <0.001
BMI turning point for 10–11 years old(K),kg/m^2^	16.4
<K, effect 1	0.035 (0.027, 0.042) <0.001
>K, effect 2	0.006 (0.005, 0.007) <0.001
BMI turning point for 12–13 years old(K),kg/m^2^	17.2
<K, effect 1	0.050 (0.038, 0.062) <0.001
>K, effect 2	0.005 (0.004, 0.006) <0.001
BMI turning point for 14–15 years old(K),kg/m^2^	20.9
<K, effect 1	0.026 (0.021, 0.030) <0.001
>K, effect 2	0.004 (0.002, 0.005) <0.001
BMI turning point for 16–17 years old(K),kg/m^2^	24.2
<K, effect 1	0.017 (0.014, 0.020) <0.001
>K, effect 2	0.000 (-0.001, 0.002) 0.621
BMI turning point for 18-19 years old(K),kg/m^2^	22
<K, effect 1	0.021 (0.015, 0.027) <0.001
>K, effect 2	0.001 (0.000, 0.003) 0.028

Age, gender, race, education level, ratio of family income to poverty, activities status, ciabetes status, ALT, ALP, AST, total calcium, total cholesterol, direct HDL cholesterol, LDL cholesterol, triglyceride, phosphorus, blood urea nitrogen, and serum glucose were adjusted.

## Discussion

Higher BMI was linked to higher total BMD in a weighted analysis involving US adolescents aged 8–19. We also performed a threshold effect analysis based on multiple regression analysis for different age groups of adolescents, and the results supported our hypothesis that the presence of a saturation value of BMI on total BMD among different age groups of adolescents could maintain a relatively healthy BMI while maintaining a higher BMD.

Several epidemiological studies in the past have demonstrated that BMD in adolescents is closely related to BMI (21–23). A cross-sectional study from Korea that included 1,063 adolescents found that BMI, lean body mass, and fat mass were all positively associated with BMD (24). Similarly, a study from Lebanon showed that obese and overweight boys had significantly higher total hip BMD and femoral neck BMD compared to boys with normal BMI (25). Our findings also demonstrate that higher BMI is associated with higher BMD in both boys and girls.

The mechanisms behind the obesity-BMD connection are unclear. One explanation is that people with obesity have a greater BMD because of the mechanical impact of their weight on their bones (26–28). Animal studies have revealed that osteocytes are particularly vulnerable to biomechanical stressors (29). They die from apoptosis in the absence of load (30), whereas when osteoblasts receive shear stress signals (31), they do not undergo apoptosis and their sclerostin secretion is inhibited (32). At the same time, osteoclast activity is slowed and osteoblast differentiation is boosted (33–35). In the population with obesity, Garnero et al. discovered a decrease in biochemical bone indicators, with a higher fall in bone resorption markers than bone production markers (36). This finding supports the theory that increased body weight causes orthostatic equilibrium. In addition to mechanical considerations, the increased BMD associated with obesity appears to be linked to estrogen activity. Estrogen has been shown to play a significant function in bone metabolism, promoting bone growth and inhibiting bone resorption (37, 38). The metabolism of estrogen and fat tissue are inextricably linked. In reality, adipose tissue is a major source of aromatase enzymes, which are needed for estrogen synthesis. Obese postmenopausal women had greater serum estrogen concentrations than non-obese women, and 17β-estradiol levels were higher in obese patients (39).

Although it is well proven that a higher BMI leads to a higher BMD, this does not mean that the risk of fracture is reduced (19). The “obesity paradox” is the name given to this phenomena (40). In children and adolescents, even being overweight has a positive effect on BMD, but the incidence of fractures is higher than in non-obese individuals (41). Preschool obesity was linked to an increased incidence of fracture in adolescents, according to a comprehensive study conducted by Lane et al. in Catalonia (42). This could be owing to excessive mechanical loading generated by extra adipose tissue (43). Whether from the perspective of reducing other systemic diseases caused by obesity or reducing the incidence of fractures in adolescents, we should find an appropriate BMI while striving for a higher BMD. Ma et al. found that, for Americans over 50 years of age, keeping the BMI at a slightly overweight value (about 26 kg/m^2^) may reduce other adverse effects while obtaining an optimal BMD (44). While in adolescents, BMI saturation values for BMD may change substantially with age, and our findings are the first to investigate BMI saturation values for BMD in US adolescents aged 8–19 years at different ages.

The mechanism of maintaining a BMI of saturation value and hence achieving optimal BMD is still unknown. Bone development trajectories and PBM are established early in life, which could explain why adult BMD does not rise after a time of restricted growth (45, 46). Another reason for BMI saturation effects is the presence of a separate bone–fat axis *in vivo* between adipose and bone tissues (47); supporting bone homeostasis and linked by numerous bioactive substances, bone and adipocytes are descended from the same stem cell parent and are competitive, according to existing research, with an increase in extra fat leading to bone loss (48). According to investigations in animal models caused by increased fat intake, BMD decreases as obesity increases in obese animals (49, 50). As a result, we hypothesized that maintaining a saturated BMI would retain enough BMD while reducing the risk of obesity-related diseases and comorbidities.

Our study has some limitations. First, this is a cross-sectional analysis; thus, temporality cannot be ascertained. Second, due to database limitations, we were unable to obtain data on participants taking calcium supplements, dietary intake of calcium, vitamin D, and lipid-lowering medications that may have an effect on BMD; therefore, our findings should be interpreted with caution. Finally, given the database limitations, we were unable to obtain a history of fractures in adolescent participants; therefore, we were unable to assess whether fracture rates were higher in adolescents with high BMI than in the general population. Despite these limitations, our study has several advantages. Because we used a nationally representative sample, our study is representative of a multi-ethnic and gender-diverse population of adolescents in the United States. In addition to this, because of the large sample size included in our study, this allowed us to divide adolescents aged 8–19 years into multiple age groups for subgroup analysis. To our knowledge, past studies have demonstrated the saturating effect of adult BMI on BMD, and the present study is the first to investigate the saturating effect of BMI on BMD in adolescents of different ages.

## Conclusion

In this study, we used multiple linear regression models, smoothed curve fitting, and saturation effect analysis models to examine the relationship between BMI and BMD in US adolescents aged 8–19 years. We found not only a simple linear positive correlation between BMI and BMD but also a saturation value that persisted across gender and age subgroups in the analysis. This work suggests that keeping BMI at saturation values may provide benefits for adolescents to maintain optimal BMD and reduce other obesity-related diseases.

## Data Availability Statement

Publicly available datasets were analyzed in this study. This data can be found here: www.cdc.gov/nchs/nhanes/.

## Ethics Statement

The studies involving human participants were reviewed and approved by NCHS Ethics Review Board. Written informed consent to participate in this study was provided by the participants’ legal guardian/next of kin.

## Author Contributions

YO and RX designed the research. YO, YQ, and XJH collected and analyzed the data. YO, CG, SX, CL, and NM drafted the manuscript. YC, XX, and RX revised the manuscript. All authors contributed to the article and approved the submitted version.

## Funding

This study was funded by the Hengyang Education Bureau Vocational Education General Project (ZJZB2020093).

## Conflict of Interest

The authors declare that the research was conducted in the absence of any commercial or financial relationships that could be construed as a potential conflict of interest.

## Publisher’s Note

All claims expressed in this article are solely those of the authors and do not necessarily represent those of their affiliated organizations, or those of the publisher, the editors and the reviewers. Any product that may be evaluated in this article, or claim that may be made by its manufacturer, is not guaranteed or endorsed by the publisher.

## Abbreviations

BMD, bone mineral density; BMI, Body Mass Index; NHANES, National Health and Nutrition Examination Survey; PBM, peak bone mass.
